# Development of a Nomogram Model for Treatment of Nonmetastatic Nasopharyngeal Carcinoma

**DOI:** 10.1001/jamanetworkopen.2020.29882

**Published:** 2020-12-11

**Authors:** Lu-Lu Zhang, Fei Xu, Di Song, Meng-Yao Huang, Yong-Shi Huang, Qi-Ling Deng, Yi-Yang Li, Jian-Yong Shao

**Affiliations:** 1 Sun Yat-Sen University Cancer Center, State Key Laboratory of Oncology in South China, Guangdong Key Laboratory of Nasopharyngeal Carcinoma Diagnosis and Therapy, Department of Molecular Diagnostics, Collaborative Innovation Center for Cancer Medicine, Guangzhou, People’s Republic of China; 2 Sun Yat-Sen University School of Mathematics, Guangzhou, People’s Republic of China; 3 Department of Oncology, First Affiliated Hospital of Guangdong Pharmaceutical University, Guangzhou, People’s Republic of China

## Abstract

**Importance:**

Because of tumor heterogeneity, overall survival (OS) differs significantly among individuals with nasopharyngeal carcinoma (NPC), even among those with the same clinical stage. Relying solely on TNM staging to guide treatment remains imperfect.

**Objectives:**

To establish a comprehensive nomogram to estimate individualized OS and to explore stratified treatment regimens for risk subgroups in nonmetastatic NPC.

**Design, Setting, and Participants:**

This cohort study included 8093 patients diagnosed with NPC at a single center in China from April 2009 to December 2015. The sample was split into a training cohort (5398 participants [66.7%]) and validation cohort (2695 [33.3%]). Data were analyzed in May 2020.

**Exposures:**

Age, T stage, N stage, Epstein-Barr virus (EBV) DNA level, serum lactate dehydrogenase (LDH) levels, and albumin (ALB) levels.

**Main Outcomes and Measures:**

The primary end point was OS. The nomogram for estimating OS was generated based on multivariate Cox proportional hazards regression. The performance of the nomogram was quantified using Harrell concordance index (*C* index), the area under the curve (AUC) of the receiver operating characteristic curve, and a calibration curve. OS rates were established using the Kaplan-Meier method, and intersubgroup differences were examined by the log-rank test.

**Results:**

Among the 8093 participants, 5688 (70.3%) were men, and the median age at diagnosis was 45 years (range, 7-85 years). Six variables (age, T stage, N stage, EBV DNA levels, LDH levels, and ALB levels) were identified through multivariate Cox regression and incorporated into a nomogram to estimate OS. The resulting nomogram showed excellent discriminative ability and significantly outperformed the eighth edition of the American Joint Committee on Cancer/Union for International Cancer Control TNM staging system for estimating OS (*C* index, 0.716 [95% CI, 0.698-0.734] vs 0.643 [95% CI, 0.624-0.661]; *P* < .001; AUC, 0.717 [95% CI, 0.698-0.737] vs 0.643 [95% CI, 0.623-0.662]; *P* < .001), and the calibration curves showed satisfactory agreement between the actual and nomogram-estimated OS rates. The validation cohort confirmed the results. Patients were stratified into 4 risk groups based on the 25th, 50th, and 75th percentile score values estimated from the nomogram. The 4 nomogram-defined risk groups demonstrated significantly different intergroup OS (3-year OS rates: risk group 1, 1328 of 1345 [98.7%]; risk group 2, 1289 of 1341 [96.1%]; risk group 3, 1222 of 1321 [92.5%]; risk group 4, 1173 of 1391 [84.3%]; *P* < .001). These risk groups were associated with the efficacy of different treatment regimens. For example, for risk group 4, induction chemotherapy with concurrent chemoradiotherapy was associated with a significantly better OS than concurrent chemoradiotherapy (log-rank *P* = .008) and intensity-modulated radiotherapy alone (log-rank *P* < .001).

**Conclusions and Relevance:**

In this study, the proposed nomogram model enabled individualized prognostication of OS and could help to guide risk-adapted treatment for patients with nonmetastatic NPC.

## Key Points

**Question:**

Could a prognostic nomogram that integrates the TNM staging system with other critical variables accurately predict overall survival and guide treatment in patients with nonmetastatic nasopharyngeal carcinoma?

**Findings:**

In this cohort study of 8093 patients with nasopharyngeal carcinoma, a nomogram was developed and validated to reliably estimate overall survival, which provided significantly better discrimination than the TNM staging system. This nomogram identified 4 risk groups with differential OS rates; these 4 risk groups were associated with the efficacy of different treatment regimens.

**Meaning:**

These finding suggest that this nomogram could enable individualized prognostication of overall survival and guide risk-adapted treatment for patients with nonmetastatic nasopharyngeal carcinoma.

## Teaser

This cohort study evaluates a comprehensive nomogram to estimate individualized overall survival among patients with nometastatic nasopharyngeal carcinoma and explores stratified treatment regimens for risk subgroups.

## Introduction

There is an unbalanced ethnic and geographic distribution in nasopharyngeal carcinoma (NPC) cases, with the highest incidences occurring in south China and Southeast Asia.^1,2^ NPC cells are highly sensitive to irradiation, which has resulted in radiotherapy (RT) becoming the mainstay treatment modality for nonmetastatic NPC.^3^ Currently, therapeutic strategies for NPC are mainly based on the American Joint Committee on Cancer/Union for International Cancer Control (AJCC/UICC) tumor-node-metastasis (TNM) staging system. National Comprehensive Cancer Network (NCCN) guidelines recommend RT alone for stage I NPC, and concurrent chemoradiotherapy (CCRT) followed by adjuvant chemotherapy (AC) or induction chemotherapy (IC) followed by CCRT for stage II to stage IVB NPC.^3^

With the application of intensity-modulated radiotherapy (IMRT), a 5-year overall survival (OS) rate of as high as 90% can be achieved for stage I NPC with RT alone.^4,5,6^ Therefore, RT was the undebated treatment modality for stage I NPC. However, whether chemotherapy is necessary for stage II NPC remains controversial in the IMRT era.^7,8,9^ There is little doubt regarding the therapeutic value of CCRT for stage III to stage IVA NPC,^10^ but studies of the efficacy of CCRT with AC have produced conflicting results.^11,12,13,14,15^ The questionable benefit, poor tolerance, and considerable toxic effects of AC have made it an increasingly less popular treatment option for stage III to stage IVA NPC.^11,16,17,18^ Compared with AC, IC is better tolerated and could eradicate micrometastases.^19^ For these reasons, IC with CCRT has become an important area of research. Three published phase 3 randomized clinical trials^20,21,22^ performed in endemic areas provide evidence that additional IC can significantly improve survival compared with treatment with CCRT alone in stage III to stage IVA NPC. With these encouraging results, the 2018 NCCN guidelines were upgraded to include the recommendation of IC with CCRT from category III to category IIA for patients with stage III to stage IVA NPC.^3^ Therefore, IC with CCRT is an increasingly important treatment regimen in managing stage III to stage IVA NPC in the IMRT era. However, there is still controversy regarding whether IC is applicable to all patients with stage III to stage IVA NPC.^20,21,22,23,24,25,26,27^

The controversy regarding the efficacy of these therapeutic strategies could be attributed to risk stratification based solely on the TNM staging system.^28^ The TNM staging system is internationally recognized, but it only describes the anatomical extent of tumors. Because of there is tumor heterogeneity, patients with equivalent TNM staging undergoing a similar intensity of treatment may have different survival outcomes.^29^ This phenomenon suggests that other factors may affect progression. Other prognostic factors may also affect survival outcomes in NPC, such as plasma Epstein-Barr virus (EBV) DNA levels,^30^ hemoglobulin (HGB) levels,^31^ serum lactate dehydrogenase (LDH) levels,^32,33^ and C-reactive protein (CRP) levels.^34^ Therefore, it is inadequate to make therapeutic decisions based solely on the TNM stage. There is an urgent need to build a more precise and comprehensive model that includes both TNM staging and other variables related to prognosis for personalized risk staging and treatment decision making.

A nomogram is a simple graphic model that incorporates multiple important factors and is a useful tool for personalized risk assessment for patients with cancer. Several nomograms have been established to guide the stratified treatment of patients with NPC of a particular TNM stage.^35,36,37,38,39^ However, to our knowledge, no comprehensive model has been developed to date to analyze the optimal stratified treatment regimen for patients with nonmetastatic NPC (stage I to stage IVA). Thus, we postulated that a prognostic nomogram that integrated the TNM staging system with other critical variables could accurately forecast OS and guide stratified treatment regimens in patients with nonmetastatic NPC in an endemic area.

## Methods

### Data Extraction and Study Population

The institutional review board and the ethics committee of Sun Yat-sen University Cancer Center (SYSUCC) approved this retrospective, anonymous analysis of data, and the requirement for written informed consent was waived. This study followed the Strengthening the Reporting of Observational Studies in Epidemiology (STROBE) reporting guideline. A dynamically updated big data intelligence platform for cancer research was developed at SYSUCC, and it incorporates comprehensive electronic health record data from routine health care data. Patients diagnosed with cancer at Sun Yat-sen University Cancer Center starting in January 2008 have been included in this platform. For each patient, data from 13 electronic health record systems were extracted. The systems included the hospital information system, the pathology system, the electronic medical record, the ultrasound system, the follow-up system, the radiation information system, the laboratory information system, the electrocardiogram system, the endoscopy system, the anesthesia information management system, the MOSAIQ radiotherapy management system, the physical examination information system, and the tumor biobank. More details regarding the big data platform are described in a previously published study.^40^ An NPC-specific database was generated by extracting data from the big data intelligence platform. A total of 8093 patients with nonmetastatic NPC who were treated with IMRT with or without chemotherapy between April 2009 and December 2015 were selected from this NPC-specific database. The inclusion criteria for our study were as follows: (1) pathologically confirmed NPC; (2) clinical stage I to IVa, according to the eighth edition of the AJCC/UICC TNM staging system; (3) received radical treatment of IMRT alone, CCRT, or IC with CCRT; (4) had available and complete pretreatment and clinical data; (5) had no subsequent second primary cancers or history of a previous malignant tumor; (6) had complete and regular follow-up data. The 8093 patients were randomly allocated into the training cohort (5398 participants [66.7%]) or validation cohort (2695 participants [33.3%]) with a 2:1 ratio using computer software–generated random numbers.

### Baseline Evaluation

Routine pretreatment assessments were performed within 2 weeks before initiation of treatment. These assessments included a complete patient history and physical examination, plasma EBV DNA load, biochemistry and hematology profiles, nasopharyngoscopy, contrast-enhanced magnetic resonance imaging (MRI) of the head and neck, chest radiography or computed tomography (CT), ultrasound or CT of the abdomen, and whole-body skeletal scintigraphy. Positron emission tomography (PET)/CT was performed if necessary. The level of plasma EBV DNA was measured using a real-time quantitative polymerase chain reaction assay (eAppendix in the Supplement), and a more detailed method for the test has been described previously.^41^ Each patient was restaged according to the eighth edition of the AJCC/UICC TNM staging system.

### Treatment Strategies

All eligible patients underwent IMRT-based radical radiotherapy. Gross tumor volumes were defined based on the MRI, CT, and PET/CT scans before initiating treatment. Delineation of the target volume was in accordance with our institutional treatment protocol and the International Commission on Radiation Units and Measurements reports 50 and 62.^42^ All patients received IMRT at a total of 66 to 72 Gy given in 28 to 33 fractions to the primary lesion, 64 to 70 Gy given in 28 to 33 fractions to the metastatic lymph node volume, 60 to 63 Gy given in 28 to 33 fractions to the high-risk clinical target volume, and 54 to 56 Gy given in 28 to 33 fractions to the low-risk clinical target volume. IMRT was delivered once daily, 5 fractions per week, during 7 weeks.

Patients included in the current study underwent radical IMRT alone, or CCRT, or IC with CCRT. The institutional treatment recommendation for NPC patients at SYSUCC is IMRT alone for stage I NPC and IC followed by CCRT or CCRT followed by AC for stage II to IVa NPC. AC was less often chosen because of limited benefit, poor compliance, and considerable toxic effects. Hence, patients treated with CCRT followed by AC were not included in this study. Reasons for not receiving recommended treatment included patients with dysfunction of vital organs, such as heart, liver, and kidney; patients who were predicted to not tolerate recommended treatment due to advanced age; and patients who refused treatment. The CCRT regimen consisted of 30 to 40 mg/m^2^ cisplatin administered weekly for a maximum of 7 cycles; 80 to 100 mg/m^2^ cisplatin administered every 3 weeks for a maximum of 3 cycles, with CCRT initiated on the first day of IMRT. The IC regimens included docetaxel (60 mg/m^2^), cisplatin (60 mg/m^2^), fluorouracil (600 mg/m^2^), docetaxel (75 mg/m^2^), and cisplatin (75 mg/m^2^) or cisplatin (80 mg/m^2^) and fluorouracil (1000 mg/m^2^), given every 3 weeks for 2 to 4 cycles.

### Clinical End Point and Follow-up

The end point of the current study was OS, defined as the time from treatment initiation to death from any cause. After treatment completion, patients returned to the hospital for review every 3 months during the first 2 years and every 6 months after that until death. Patients were followed up via telephone if their recent examination records were not available. Follow-up duration was defined as the date of treatment initiation until last contact or death. Posttreatment surveillance at each follow-up appointment included a comprehensive physical examination, plasma EBV DNA load, nasopharyngoscopy, and imaging assessment similar to the pretreatment examinations. Clinical suspicion of treatment failure was confirmed based on imaging assessment with or without cytological biopsies.

### Statistical Analysis

SPSS statistical software version 23.0 (IBM Corp) and R software version 3.4.4 (R Project for Statistical Computing) were used for all statistical analyses. We analyzed the following baseline prognostic covariates: sex, age, T stage, N stage, World Health Organization histologic type, family history of cancer, alcohol consumption, cigarette consumption, plasma EBV DNA load, HGB level, LDH level, ALB level, and CRP level. The continuous variable age was divided into 2 subgroups based on the median value, and other continuous variables were converted into categorical variables based on generally recognized cutoff values. The cutoff value of EBV DNA load (2000 copies/mL),^30^ HGB (120 copy/mL),^43^ LDH (245 U/L),^33,43^ ALB (4.0 g/dL [to convert to grams per liter, multiply by 10])^44^ and CRP (0.1 mg/dL and 0.3 mg/dL [to convert to grams per liter, multiply by 10])^34^ were demonstrated to have powerful prognostic value in previous research.

To compare the differences of categorical variables in the training and validation cohort, the χ^2^ test or Fisher exact test were used. OS rates were analyzed using the Kaplan-Meier method, and intersubgroup differences were examined by the log-rank test. Univariate and multivariate analyses using Cox proportional hazard regression models were used to test the significance of independent risk factors. Variables with *P* < .05 in the univariate analysis were entered into the multivariate analysis to estimate the significance of each variable. Hazard ratios (HRs) and 95% CIs were calculated as the summary statistics. Moreover, forest plots were generated to visualize the HRs and 95% CIs of the potential baseline prognostic covariates for OS.

We aimed to establish a practical model for the individualized estimation of OS in nonmetastatic NPC. Nomograms to estimate 3-year and 5-year OS were generated based on the independent risk factors established by multivariate regression analysis using R version 3.4.4. The performance of the nomogram to discriminate and calibrate was first measured in the training cohort using Harrell concordance index (*C* index), the area under the curve (AUC) of the receiver operating characteristic (ROC) curve, and calibration curve. Then the nomogram was validated in the validation cohort. The value of both the *C* index and AUC ranged from 0.5 to 1.0. The maximum *C* index or AUC of 1.0 indicates a perfect discrimination ability of the nomogram model, and a *C *index or AUC value of at least 0.7 indicated that the model has a good discriminating ability. Consistency between nomogram-estimated 3-year and 5-year OS probabilities and actual outcome were assessed, with the 45-degree line representing the ideal reference line. Comparisons between nomogram models, TNM staging system, and single prognostic factors were made using rcorrp.cens in the hmisc package in R. By grouping patients into 4 risk groups based on the nomogram-defined scores, we conducted a subgroup survival analysis and investigated the optimal treatment in those 4 subgroups. A 2-tailed *P* < .05 was considered statistically significant.

## Results

### Baseline Characteristics and Survival

The baseline clinical and treatment characteristics of the 8093 eligible patients with nonmetastatic NPC are shown in eTable 1 in the Supplement, and the characteristics were well balanced between the training cohort and validation cohort. Among the 8093 participants, 5688 (70.3%) were men, and the median age at diagnosis was 45 years (range, 7-85 years). Most patients received IC with CCRT (3566 [44.1%]) or CCRT (3388 [41.9%]), while 1139 (14.1%) underwent IMRT alone. The median follow-up time was 53.2 months (range, 2.0-111.1 months) for the training cohort and 53.2 months (range, 1.7-106.0 months) for the validation cohort. The 3-year and 5-year OS rates were 5012 (92.8%) and 4680 (86.7%), respectively, in the training cohort and 2493 (92.5%) and 2342 (86.9%), respectively, in the validation cohort. At the date of the last follow-up in the training cohort, 643 patients (11.9%) had experienced local-regional recurrence and 518 (9.6%) experienced distant metastasis; in the validation cohort, 248 patients (9.2%) had experienced local-regional recurrence and 345 (12.8%) experienced distant metastasis. Overall, 686 patients (12.7%) in the training cohort and 339 (12.6%) in the validation cohort had died from any cause.

### Independent Prognostic Factors Associated With Overall Survival

Univariate analysis indicated that older age; male gender; World Health Organization histologic type I to II; advanced T stage; advanced N stage; cigarette consumption; increased EBV DNA, LDH, and CRP levels; and decreased ALB levels were significantly associated with worse OS in the training cohort. Older age; male gender; World Health Organization histologic type I to II; advanced T stage; advanced N stage; cigarette consumption; family history of cancer; increased EBV DNA, LDH and CRP levels; and decreased HGB and ALB levels were significantly associated with worse OS in the validation cohort. The forest plot showing the results of these univariate analyses is presented in eFigure 1 in the Supplement.

In the multivariate analysis, age; T stage; N stage; and EBV DNA, LDH, and ALB levels were independent risk factors in the training cohort. In the validation cohort, age; World Health Organization histologic type; T stage; N stage; and EBV DNA, HGB, and LDH levels were significantly associated with OS. The detailed results of the multivariate analyses are summarized in eTable 2 in the Supplement.

### Nomogram Development and Validation

**Figure 1.**
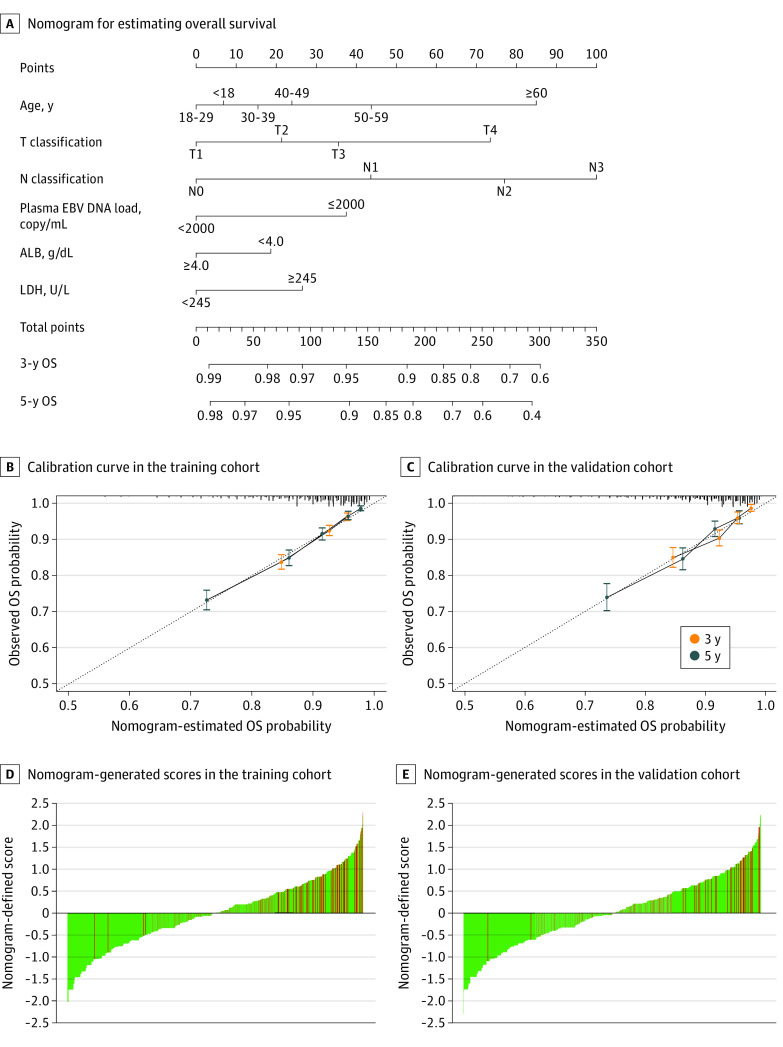
Nomogram, Calibration Curves, and Nomogram-Generated Scores Red and green bars represent the scores for individual patients who did and did not die, respectively. ALB indicates albumin; EBV, Epstein-Barr virus; LDH, lactate dehydrogenase; OS, overall survival.

We built the OS prognostic nomogram based on the resulting coefficients from the multivariate Cox analysis in the training cohort (Figure 1A). Calibration curves for OS estimation revealed an excellent agreement between the OS estimates derived from the nomogram and the actual OS probabilities in the 2 cohorts (Figure 1B). Within those 6 variables that construct the nomogram, each model covariate was assigned a score by drawing a vertical line straight down to the axis labeled points. By summing the total score and locating it on the total points scale, the individual probabilities of 3-year or 5-year OS can be determined. The nomogram-generated scores for each patient are depicted in Figure 1C. The *C* indices of the nomogram for OS estimates were 0.716 (95% CI, 0.698-0.734) and 0.713 (95% CI, 0.688-0.738) in the training and validation cohorts, respectively.

For the training and validation cohorts, the *C* indices of the constructed nomogram significantly outperformed the eighth edition of the AJCC/UICC TNM staging system (training cohort: 0.643; 95% CI, 0.624-0.661; *P* < .001; validation cohort: 0.640; 95% CI, 0.613-0.667; *P* < .001) and the single prognostic variables age (training cohort: 0.588; 95% CI, 0.566-0.510; *P* < .001; validation cohort: 0.582; 95% CI, 0.550-0.615; *P* < .001), T stage (training cohort: 0.613; 95% CI, 0.593-0.633; *P* < .001; validation cohort: 0.606; 95% CI, 0.578-0.635; *P* < .001), N stage (training cohort: 0.630; 95% CI, 0.610-0.650; *P* < .001; validation cohort: 0.607; 95% CI, 0.578-0.637; *P* < .001), EBV DNA level (training cohort: 0.617; 95% CI, 0.599-0.635; *P* < .001; validation cohort: 0.624; 95% CI, 0.599-0.649; *P* < .001), LDH level (training cohort; 0.536; 95% CI, 0.522-0.550; *P* < .001; validation cohort: 0.542; 95% CI, 0.522-0.562; *P* < .001), and ALB level (training cohort 0.532; 95% CI, 0.518-0.545; *P* < .001; validation cohort: 0.531; 95% CI, 0.512-0.551; *P* < .001) for estimating OS in nonmetastatic NPC (Table).

**Table.** Summary of the *C* Index of Prognostic Models and Single Risk Factors for Overall Survival in Patients With Nonmetastatic Nasopharyngeal Carcinoma

Model	*C* index (95% CI)		*P* value
	Training cohort	Validation cohort	
Prognostic models			
Proposed nomogram	0.716 (0.698-0.734)	0.713 (0.688-0.738)	NA
Eighth TNM stage	0.643 (0.624-0.661)	0.640 (0.613-0.667)	<.001
Single risk factors			
Age	0.588 (0.566-0.610)	0.582 (0.550-0.615)	<.001
T stage	0.613 (0.593-0.633)	0.606 (0.578-0.635)	<.001
N stage	0.630 (0.610-0.650)	0.607 (0.578-0.637)	<.001
EBV DNA	0.617 (0.599-0.635)	0.624 (0.599-0.649)	<.001
LDH	0.536 (0.522-0.550)	0.542 (0.522-0.562)	<.001
ALB	0.532 (0.518-0.545)	0.531 (0.512-0.551)	<.001

Abbreviations: ALB, albumin; EBV, Epstein-Barr virus; eighth TNM stage, eighth edition of the American Joint Committee on Cancer/Union for International Cancer Control staging system; LDH, lactate dehydrogenase; NA, not applicable; TNM, tumor-node-metastasis.

Moreover, to further assess the discriminatory performance of the constructed nomogram, ROC curves were generated to evaluate the prognostic performance of the nomogram, TNM stage, and the 6 single variables. This analysis revealed that the AUCs of the nomogram statistically significantly outperformed the eighth edition of the AJCC/UICC TNM staging system and 6 variables for OS estimation (eg, training cohort, nomogram: 0.717 [95% CI, 0.698-0.737]; TNM staging system: 0.643 [95% CI, 0.623-0.662]; *P* < .001; validation cohort, nomogram: 0.714 [95% CI, 0.687-0.741]; TNM staging system: 0.636 [95% CI, 0.606-0.666); *P* < .001). The detailed results of the AUCs are summarized in eFigure 2 in the Supplement.

### Construction of 4 Distinct Risk Groups to Estimate Differential Prognosis

**Figure 2.**
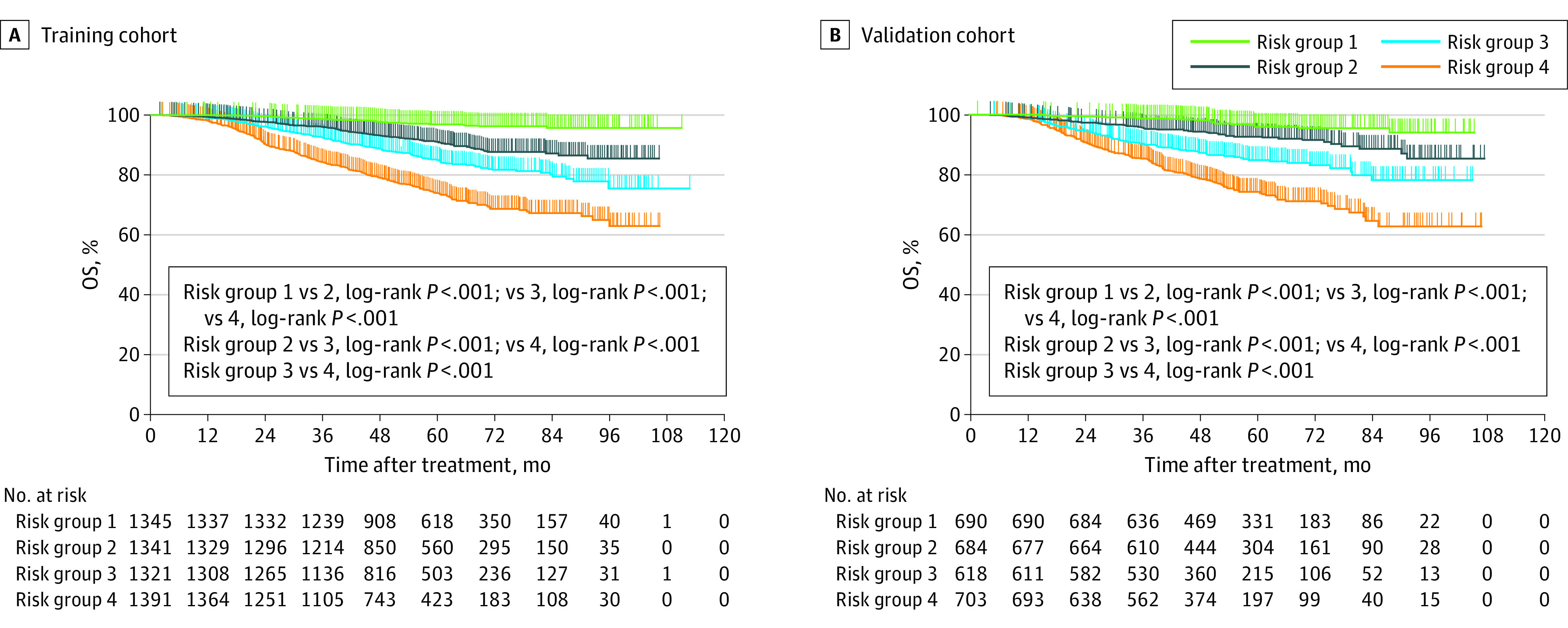
Kaplan-Meier Curves for Patients with Nonmetastatic Nasopharyngeal Carcinoma Based on Risk Group Stratification in the Training and Validation Cohorts Patients were stratified into 4 risk groups based on the 25th percentile (score, 2.7), 50th percentile (score, 3.3), and 75th percentile (score, 3.8) score values estimated from the constructed nomogram.

Patients in the 2 cohorts were stratified into 4 risk groups based on the 25th percentile (score, 2.7), 50th percentile (score, 3.3), and 75th percentile (score, 3.8) score values estimated from the established nomogram. Accordingly, 1345 patients (24.9%), 1341 patients (24.8%), 1321 patients (24.5%), and 1391 patients (25.8%) in the training cohort, and 690 patients (25.6%), 684 patients (25.4%), 618 patients (22.9%), and 703 patients (26.1%) in the validation cohort were classified in risk groups 1 to 4, respectively. The number of events in each group is listed in eTable 3 in the Supplement. The subgroup survival analysis revealed that the 3-year and 5-year OS rates gradually lowered from risk group 1 to risk group 4 in the training cohort (3-year OS rates: risk group 1, 1328 [98.7%]; risk group 2, 1289 [96.1%]; risk group 3, 1222 [92.5%]; risk group 4, 1173 [84.3%]; *P* < .001; 5-year OS rates: risk group 1, 1303 [96.9%]; risk group 2, 1222 [91.1%]; risk group 3, 1124 [85.1%]; risk group 4, 1029 [74.0%]; *P* < .001), and this result was verified in the validation cohort (eTable 4 in the Supplement). Kaplan-Meier survival curves for OS are shown in Figure 2.

### Optimal Treatment Regimens Based on the 4 Established Risk Groups

**Figure 3.**
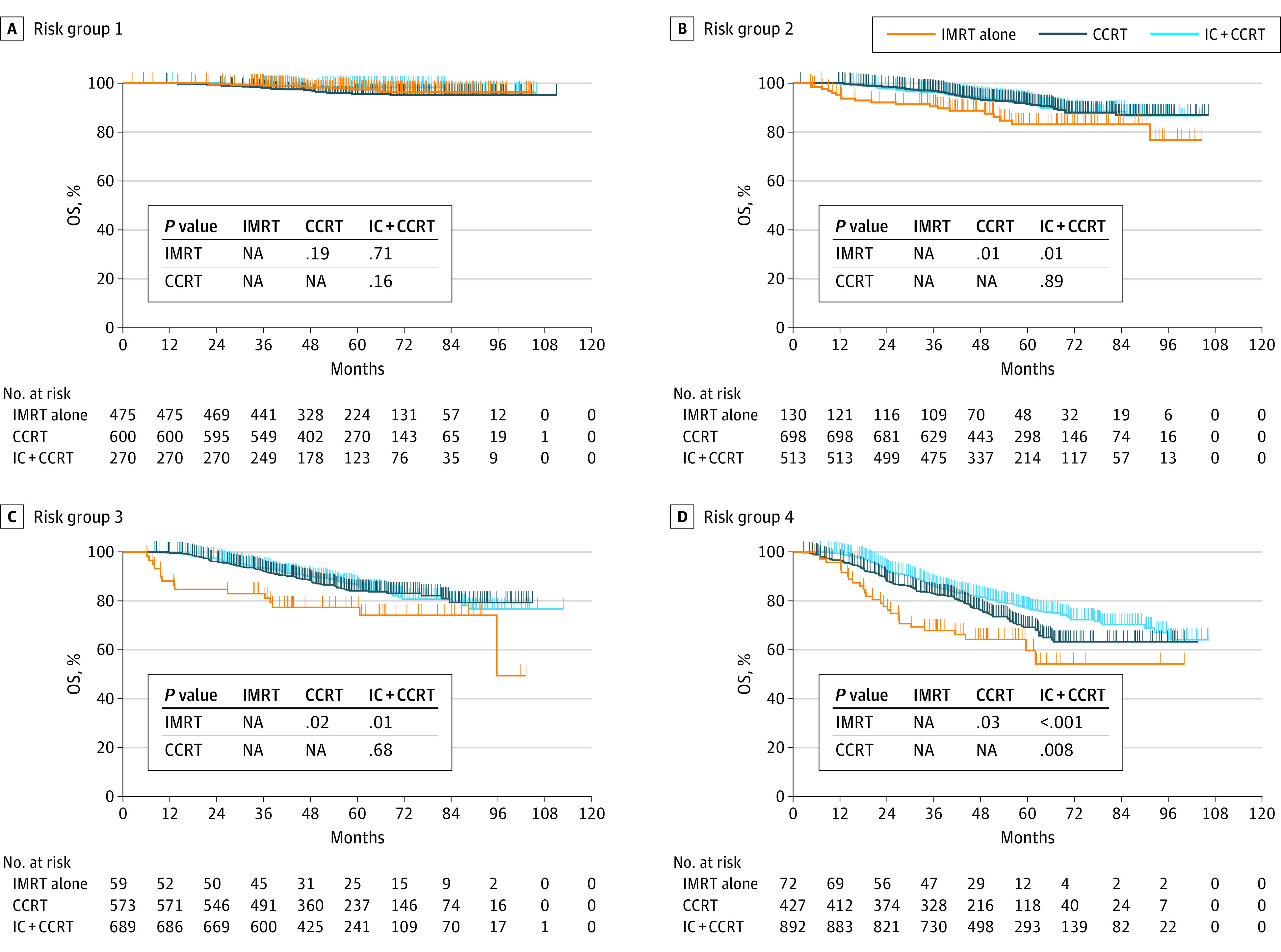
Optimal Treatment Regimens Based on Nomogram-Defined Risk Groups in the Training Cohort Patients were stratified into 4 risk groups based on the 25th percentile (score, 2.7), 50th percentile (score, 3.3), and 75th percentile (score, 3.8) score values estimated from the constructed nomogram. The log-rank test was used to compare the survival rate and calculate the *P* value. CCRT indicates concurrent chemoradiotherapy; IC, induction chemotherapy; IMRT, intensity-modulated radiation therapy.

**Figure 4.**
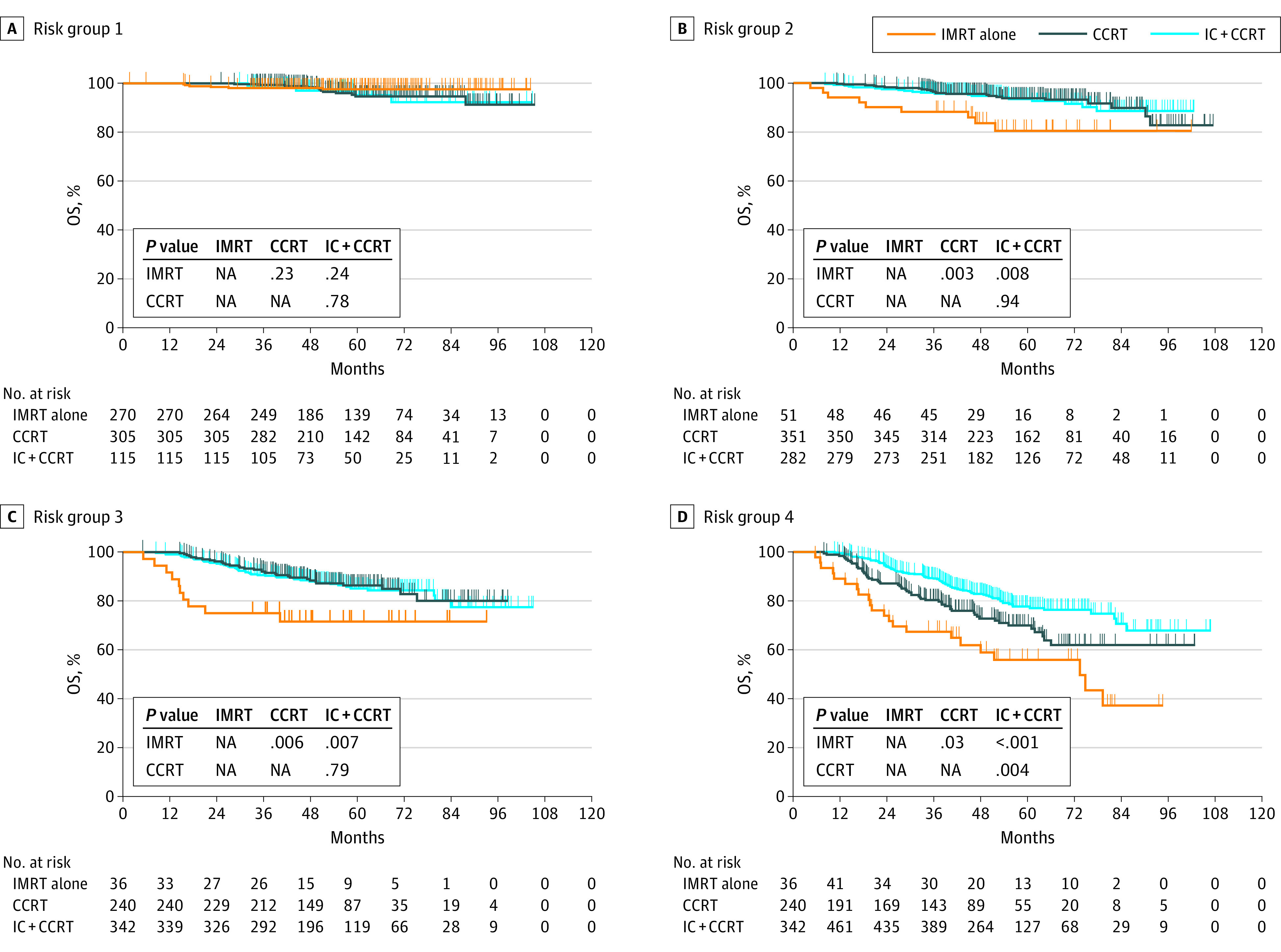
Optimal Treatment Regimens Based on Nomogram-Defined Risk Groups in the Validation Cohort Patients were stratified into 4 risk groups based on the 25th percentile (score, 2.7), 50th percentile (score, 3.3), and 75th percentile (score, 3.8) score values estimated from the constructed nomogram. The log-rank test was used to compare the survival rate and calculate the *P* value. CCRT indicates concurrent chemoradiotherapy; IC, induction chemotherapy; IMRT, intensity-modulated radiation therapy.

We explored the optimal treatment regimens for each risk group by comparing the efficacy of different treatment modalities. The number of patients receiving different treatment regimens within nomogram-defined risk groups are shown in eTable 5 in the Supplement. Within risk group 1 (Figure 3A and Figure 4A), a nonsignificant difference in OS was observed for IC with CCRT (training cohort, log-rank *P* = .71; validation cohort, log-rank *P* = .24) or CCRT (training cohort, log-rank *P* = .19; validation cohort, long rank *P* = .23) compared with IMRT alone.

For risk group 2 (Figure 3B and Figure 4B), IC with CCRT (training cohort, log-rank *P* = .01; validation cohort: log-rank *P* = .008) and CCRT (training cohort, log-rank *P* = .01; validation cohort, log-rank *P* = .003) were superior to IMRT alone in terms of OS, but OS was comparable between the IC with CCRT and CCRT groups (training cohort, log-rank *P* = .89; validation cohort, log-rank *P* = .94). The results for risk group 3 (Figure 3C and Figure 4C) were similar to those in risk group 2. Patients who received IC with CCRT (training cohort, log-rank *P* = .01; validation cohort, log-rank *P* = .007) or CCRT (training cohort, log-rank *P* = .02; validation cohort, log-rank *P* = .006) had statistically improved OS compared with those who received IMRT alone, but patients who received IC with CCRT did not show significantly better OS compared with those who received CCRT (training cohort, log-rank *P* = .68; validation cohort, log-rank *P* = .79).

Finally, for risk group 4 (Figure 3D and Figure 4D), IC with CCRT was associated with a significantly better OS than CCRT (training cohort, log-rank *P* = .008; validation cohort, log-rank *P* = .004). It was also associated with a statistically significant better OS than IMRT alone (training cohort, log-rank *P* < .001; validation cohort, log-rank *P* < .001).

## Discussion

Tumor heterogeneity necessitates personalized cancer therapy. Determining whether an individual patient is at high risk of adverse clinical outcomes may help oncologists develop the most appropriate personalized treatment regimen. However, there are still controversies regarding optimal treatment regimens.^7,8,9,10,11,12,13,14,15,16,17,18,19,20,21,22,23,24,25,26,27,28^ In part, this may be because of the use of the TNM staging system for risk stratification, which remains imperfect due to its simplicity and the heterogeneity of prognosis within patients with the same stage.

To our knowledge, this is the first study to establish and validate a comprehensive nomogram to estimate OS that combines the TNM staging system and other widely assessed clinical characteristics to accurately assess the optimal stratified treatment regimens in patients with nonmetastatic NPC. The resulting nomogram showed excellent discriminative ability (training cohort: *C* index, 0.716; 95% CI, 0.698-0.734; validation cohort: *C* index, 0.713; 95% CI, 0.688-0.738) with excellent agreement between actual OS and nomogram-estimated OS probabilities, as confirmed by the calibration curve. This demonstrates that the nomogram represents a reasonable model with a powerful prognostic performance to estimate OS in nonmetastatic NPC. In addition, the discriminatory ability of this nomogram was significantly better than that of the traditional TNM staging and other clinical variables. A series of nomograms in the published literature is available to predict survival in NPC,^34,35,36,37,38,39,45,46,47,48^ but unfortunately, most of these were not analyzed for their role in guiding treatment choice. Previously, only 5 studies have established nomogram models for facilitating personalized patient treatment strategies within a particular TNM stage.^35,36,37,38,39^ For example, Sun et al^36^ established a nomogram to estimate OS and guide individual treatment for patients with locally recurrent NPC. Zhang et al^39^ developed a nomogram for stage III to stage IVA NPC patients to select a subgroup of patients who may benefit from additional IC compared with CCRT alone. This nomogram could serve as a facilitator of individual treatment. However, to our knowledge, a prognostic nomogram for nonmetastatic NPC has never been developed as a tool to help guide treatment decisions.

To address this unmet need, we adopted a real-world large-scale study that enrolled 8093 patients with nonmetastatic NPC to construct and validate a nomogram for OS estimation and to guide personalized treatment. In a multivariate analysis, we found that age, T stage, N stage, EBV DNA levels, LDH levels, and ALB levels should be incorporated in the nomogram model. In agreement with our findings, these 6 variables have been previously reported to be associated with survival in NPC.^30,32,33,34,49^ Based on nomogram-generated scores, patients were classified into 4 risk groups to estimate OS. Importantly, those 4 risk groups demonstrated significantly different intergroup prognoses in terms of OS. Accordingly, the 4 risk groups were found to each have an optimal treatment regimen. Within risk group 1, a nonsignificant difference in OS was observed for IC with CCRT or CCRT compared with IMRT alone; for risk group 2 and 3, IC with CCRT and CCRT were superior to IMRT alone in terms of OS, but OS was comparable between the IC with CCRT and CCRT groups; within risk group 4, IC with CCRT was associated with better OS than CCRT or IMRT alone. Through this model, an oncologist could accurately and efficiently estimate a patient’s prognosis and guide the development of an individualized treatment regimen.

It is worth mentioning that this study did not include patients who received CCRT with AC. Although CCRT followed by AC was recommended by the NCCN guideline for stage II to stage IVA NPC,^3^ the efficacy of CCRT with AC has been debated.^11,12,13,14,15,16^ A phase 3 multicenter, randomized clinical trial conducted by Chen et al^13^ suggested that the addition of AC to standard CCRT failed to significantly improve survival and led to an increased incidence of acute toxic effects. The potential reason for the limited benefit of AC may be poor tolerance of the adjuvant phase, with a compliance rate of 55% to 75% and with 23% to 43% of patients experiencing grade 3 to 4 toxic effects.^11,16,17,18^ CCRT with AC was less often selected in our cohort because of the limited benefit, poor adherence, and considerable toxic effects. Therefore, patients treated with CCRT with AC were not included in our current study.

### Limitations

The present study has several limitations that must be acknowledged. First, a selection bias might be inevitable because of the retrospective nature of this study. However, we have included relatively large training and validation cohorts to construct the nomogram and internally validated it because this may reduce the bias caused by the retrospective data analysis. Second, even though the internal validation cohort showed excellent discriminative ability with a satisfactory agreement between the actual OS and the nomogram-estimated OS probabilities as confirmed by the calibration curve, we did not perform external validation. Hence, additional validation of our results with large-scale data in other centers is necessary. Third, this study was performed in an endemic area and therefore may not be generalizable to different patient populations with NPC. Fourth, this study predated gemcitabine/cisplatin as an induction regimen, which is commonly used following randomized data supporting this combination.

## Conclusions

This study established and validated a nomogram that integrated multiple biomarkers to estimate OS and potentially forecast the optimal stratified treatment regimens in patients with nonmetastatic NPC. This nomogram successfully identified 4 distinct risk groups with differential OS rates and provided significantly better discrimination than the eighth edition of AJCC/UICC TNM staging system. These 4 risk groups were associated with the efficacy of different treatment regimens. This nomogram could be used as an important prognostic tool to assist in making medical decisions in combination with other clinical factors.

## Notes

Supplement. **eAppendix. **Supplementary Methods **eTable 1. **Clinicopathologic Characteristics of 8093 Patients with Nonmetastatic Nasopharyngeal Carcinoma **eTable 2. **Multivariate Cox Proportional Hazard Regression Analysis of Independent Variables for OS in Nonmetastatic Nasopharyngeal Carcinoma **eTable 3. **Number of Events in the Nomogram-Defined Risk Groups **eTable 4. **Survival of the Nomogram-Defined Risk Groups **eTable 5. **Number of Patients Receiving Different Treatment Regimens Within Nomogram-Defined Risk Groups **eFigure 1. **Forest Plot Showing Univariate Cox Proportional Hazard Regression Analysis of Potential Risk Factors for Overall Survival in Nonmetastatic Nasopharyngeal Carcinoma **eFigure 2. **Receiver Operating Characteristic (ROC) Curves
