# Supplementary material for: Development of a Nomogram Model for Treatment of Nonmetastatic Nasopharyngeal Carcinoma
Source: JAMA Netw Open. 2020 Dec 11;3(12):e2029882. doi: 10.1001/jamanetworkopen.2020.29882 (PMC7733160; doi:10.1001/jamanetworkopen.2020.29882)
Supplement: Supplement. — eAppendix. Supplementary Methods eTable 1. Clinicopathologic Characteristics of 8093 Patients with Nonmetastatic Nasopharyngeal Carcinoma eTable 2. Multivariate Cox Proportional Hazard Regression Analysis of Independent Variables for OS in Nonmetastatic Nasopharyngeal Carcinoma eTable 3. Number of Events in the Nomogram-Defined Risk Groups eTable 4. Survival of the Nomogram-Defined Risk Groups eTable 5. Number of Patients Receiving Different Treatment Regimens Within Nomogram-Defined Risk Groups eFigure 1. Forest Plot Showing Univariate Cox Proportional Hazard Regression Analysis of Potential Risk Factors for Overall Survival in Nonmetastatic Nasopharyngeal Carcinoma eFigure 2. Receiver Operating Characteristic (ROC) Curves [file jamanetwopen-e2029882-s001.pdf]

Supplemental Online Content

Zhang LL, Xu F, Song D, et al. Development of a nomogram model for treatment of nonmetastatic nasopharyngeal carcinoma. *JAMA Netw Open*. 2020;3(12):e2029882. doi:10.1001/jamanetworkopen.2020.29882

eAppendix. Supplementary Methods

eTable 1. Clinicopathologic Characteristics of 8093 Patients with Nonmetastatic Nasopharyngeal Carcinoma

eTable 2. Multivariate Cox Proportional Hazard Regression Analysis of Independent Variables for OS in Nonmetastatic Nasopharyngeal Carcinoma

eTable 3. Number of Events in the Nomogram-Defined Risk Groups

eTable 4. Survival of the Nomogram-Defined Risk Groups

eTable 5. Number of Patients Receiving Different Treatment Regimens Within Nomogram-Defined Risk Groups

eFigure 1. Forest Plot Showing Univariate Cox Proportional Hazard Regression Analysis of Potential Risk Factors for Overall Survival in Nonmetastatic Nasopharyngeal Carcinoma

eFigure 2. Receiver Operating Characteristic (ROC) Curves

This supplemental material has been provided by the authors to give readers additional information about their work.

© 2020 Zhang LL et al. *JAMA Network Open.*

eAppendix. Supplementary Methods

Pre-treatment plasma EBV DNA sampling and assay

Blood samples were collected within two weeks before treatment. To separate the plasma and peripheral blood cells, the blood were placed in an ethylene diaminete traacetic acide tube and centrifuged at 1600 g for 15 minutes. Viral DNA were extracted using the QIAamp Blood Kit (Qiagen, Hilden, Germany). EBV DNA levels were measured using a real-time PCR assay toward the BamHI-W region of the EBV genome[1]. The sequences of the forward and reverse primers used in the assay were 5`-GCCAG AGGTA AGTGG ACTTT-3` and 5`-TACCA CCTCC TCTTC TTGCT-3`. The dual fluorescently-labelled oligomer of 5`-(FAM) CACAC CCAGG CACAC ACTAC ACAT (TAMRA)-3` served as the probe.

1. Shao JY, Li YH, Gao HY, et al. Comparison of plasma Epstein-Barr virus (EBV) DNA levels and serum EBV immunoglobulin A/virus capsid antigen antibody titers in patients with nasopharyngeal carcinoma. Cancer 2004, 100(6):1162-1170.

© 2020 Zhang LL et al. *JAMA Network Open.*

eTable 1. Clinicopathologic Characteristics of 8093 Patients with Nonmetastatic Nasopharyngeal Carcinoma

Characteristic	Training cohort (n= 5,398)	Validation cohort (n=2,695)	*P* Value[†]
**Age (years)**			0.866
**< 18**	41 (0.8)	23 (0.9)	
**18–29**	401 (7.4)	208 (7.7)	
**30–39**	1,258 (23.3)	646 (24.0)	
**40–49**	1,881 (34.8)	908 (33.7)	
**50–59**	1,213 (22.5)	619 (23.0)	
**≥60**	604 (11.2)	291 (10.8)	
**Gender**			< 0.001
**Male**	3,941 (73.0)	1,747 (64.8)	
**Female**	1,457 (27.0)	948 (35.2)	
**WHO histologic type**			0.871
**Type I-II**	131 (2.4)	67 (2.5)	
**Type III**	5,267 (97.6)	2,628 (97.5)	
**T stage***			< 0.001
**T1**	942 (17.5)	243 (9.0)	
**T2**	860 (15.9)	495 (18.4)	
**T3**	2,544 (47.1)	1,292 (47.9)	
**T4**	1,052 (19.5)	665 (24.7)	
**N stage***			< 0.001
**N0**	874 (16.2)	333 (12.4)	
**N1**	2,747 (50.9)	1,181 (43.8)	
**N2**	1,127 (20.9)	8,67 (32.2)	
**N3**	650 (12.0)	314 (11.7)	
**TNM stage***			0.180
**I**	339 (6.3)	166 (6.2)	

© 2020 Zhang LL et al. *JAMA Network Open.*

**II**	973 (18.0)	486 (18.0)	
**III**	2,515 (46.6)	1,198(44.5)	
**IV**	1,571 (29.1)	845 (31.4)	
**Cigarette consumption**			0.846
**No**	3,511 (65.0)	1,747 (64.8)	
**Yes**	1,887 (35.0)	948 (35.2)	
**Alcohol consumption**			0.118
**No**	4,620 (85.6)	2,341 (86.9)	
**Yes**	778 (14.4)	354 (13.1)	
**Family of cancer history**			0.672
**No**	3,940 (73.0)	1,979 (73.4)	
**Yes**	1,458 (27.0)	716 (26.6)	
**EBV DNA, copy/mL**			0.404
**< 2000**	2,737 (50.7)	1,393 (51.7)	
**≥ 2000**	2,661 (49.3)	1,302 (48.3)	
**HGB, g/L**			0.360
**<120**	363 (6.7)	196 (7.3)	
**≥120**	5,035 (93.3)	2,499 (92.7)	
**LDH, U/L**			0.164
**<245**	4,981 (92.3)	2,510 (93.1)	
**≥245**	417 (7.7)	185 (6.9)	
**ALB, g/L**			0.703
**<40**	469 (8.7)	241 (8.9)	
**≥40**	4,929 (91.3)	2,454 (91.1)	
**CRP, mg/L**			0.508
**<1.0**	1,785 (33.1)	863 (32.0)	
**1.0-3.0**	2,015 (37.3)	1,004 (37.3)	

© 2020 Zhang LL et al. *JAMA Network Open.*

**≥3.0**	1,598 (29.6)	828 (30.7)	
**Chemotherapy**			0.109
**IMRT alone**	736 (13.6)	403 (15.0)	
**CCRT**	2,298 (42.6)	1,090 (40.4)	
**IC+CCRT**	2,364 (43.8)	1,202 (44.6)	

Statistical comparisons were computed using the Chi-square test, two-tailed *P* values < 0.05 were considered statistically significant.* According to the 8th edition of the AJCC/UICC staging system. **Abbreviations**: WHO = World Health Organization; EBV DNA = Epstein-Barr virus deoxyribonucleic acid; HGB = hemoglobin; LDH = lactate dehydrogenase; ALB = albumin; CRP = C-reactive protein; IMRT = intensity-modulated radiotherapy; CCRT = concurrent chemoradiotherapy; IC = induction chemotherapy.

© 2020 Zhang LL et al. *JAMA Network Open.*

eTable 2. Multivariate Cox Proportional Hazard Regression Analysis of Independent Variables for OS in Nonmetastatic Nasopharyngeal Carcinoma

**Risk factors**	**Training cohort**		**Validation cohort**	
	**HR (95% CI)**	***P***	**HR (95% CI)**	***P***
**Age, years**		< 0.001		< 0.001
< 18 vs.≥60	0.370 (0.162-0.843)	0.018	0.287 (0.070-1.175)	0.083
18–29 vs. ≥60	0.331 (0.231-0.476)	< 0.001	0.244 (0.144-0.415)	< 0.001
30–39 vs. ≥60	0.398 (0.312-0.507)	< 0.001	0.370 (0.265-0.517)	< 0.001
40–49 vs. ≥60	0.440 (0.355-0.545)	< 0.001	0.434 (0.321-0.587)	< 0.001
50–59 vs. ≥60	0.577 (0.462-0.722)	< 0.001	0.409 (0.296-0.556)	< 0.001
**Gender (*Female vs Male*)**	0.818 (0.667-1.004)	0.055	0.742 (0.541-1.018)	0.065
**WHO histologic type (*Type III vs. Type I-II*)**	0.700 (0.482-1.018)	0.062	0.442 (0.261-0.748)	0.002
**T stage**		< 0.001		< 0.001
(T2 vs. T1)	1.352(0.963-1.900)	0.082	2.138 (1.283-3.561)	0.004
(T3 vs. T1)	1.600 (1.198-2.137)	0.001	2.313 (1.447-3.696)	< 0.001
(T4 vs. T1)	2.654 (1.961-3.590)	< 0.001	3.158 (1.948-5.120)	< 0.001
**N stage**		< 0.001		< 0.001
(N1 vs. N0)	1.806 (1.293-2.524)	0.001	1.208 (0.800-1.825)	0.368
(N2 vs. N0)	2.789 (1.965-3.960)	< 0.001	1.535 (0.988-2.382)	0.056
(N3 vs. N0)	3.773 (2.622-5.428)	< 0.001	2.217 (1.397-3.516)	0.001
**Cigarette consumption (*Yes vs. No*)**	1.120 (0.944-1.329)	0.194	1.136 (0.892-1.448)	0.301
**Family of cancer history (*Yes vs. No*)**	/	/	0.788 (0.606-1.024)	0.074
**EBV DNA, copy/mL (≥ *2000 vs. < 2000*)**	1.635 (1.368-1.955)	< 0.001	2.005 (1.554-2.586)	< 0.001
**HGB, g/L (≥ *120 vs. <120*)**	/	/	0.596 (0.402-0.883)	0.010
**LDH, U/L (≥ *245 vs. <245*)**	1.429 (1.143-1.786)	0.002	1.787 (1.293-2.469)	< 0.001
**ALB, *g/L(≥40 vs.<40)***	0.780 (0.621-0.980)	0.033	0.808 (0.588-1.110)	0.188

© 2020 Zhang LL et al. *JAMA Network Open.*

**CRP**		0.217		0.936
***1.0-3.0 vs.* <1.0**	0.962 (0.792-1.170)	0.700	1.049 (0.792-1.389)	0.738
≥3.0 vs. <1.0	1.123 (0.923-1.366)	0.248	1.013 (0.760-1.351)	0.929

Abbreviations: HR = Hazard ratio; CI= confidence intervals; WHO = World Health Organization; EBV DNA = Epstein-Barr virus deoxyribonucleic acid; HGB = hemoglobin; LDH = lactate dehydrogenase; ALB = albumin; CRP = C-reactive protein.

© 2020 Zhang LL et al. *JAMA Network Open.*

eTable 3. Number of Events in the Nomogram-Defined Risk Groups

Number of events	Risk group 1 (n = 1,345,%)	Risk group 2 (n = 1,341,%)	Risk group 3 (n = 1,321,%)	Risk group 4 (n = 1,391,%)
**Training cohort (n = 5,398)**	38 (2.8)	119 (8.9)	188 (14.2)	341 (24.5)
**Validation cohort (n = 2,695)**	23 (3.3)	53 (7.7)	90 (14.6)	173 (24.6)

© 2020 Zhang LL et al. *JAMA Network Open.*

eTable 4. Survival of the Nomogram-Defined Risk Groups

**Nomogram-defined subgroups**	**3-year Overall Survival**		**5-year Overall Survival**	
	**Training cohort**	**Validation cohort**	**Training cohort**	**Validation cohort**
**Risk group 1**	98.7%	98.7%	96.9%	95.9%
**Risk group 2**	96.1%	95.7%	91.1%	92.7%
**Risk group 3**	92.5%	90.2%	85.1%	84.4%
**Risk group 4**	84.3%	85.3%	74.0%	74.2%
***P* values**	< 0.001	< 0.001	< 0.001	< 0.001

© 2020 Zhang LL et al. *JAMA Network Open.*

eTable 5. Number of Patients Receiving Different Treatment Regimens Within Nomogram-Defined Risk Groups

Abbreviations: IMRT = intensity-modulated radiotherapy; CCRT = concurrent chemoradiotherapy; IC

Number of Patients		Risk group 1 (n,%)	Risk group 2 (n,%)	Risk group 3 (n,%)	Risk group 4 (n,%)
Training cohort (n = 5,398)	IMRT alone	475 (35.3)	130 (9.7)	59 (4.5)	72 (5.2)
	CCRT	600 (44.6)	698 (52.1)	573 (43.4)	427 (30.7)
	IC+CCRT	270 (20.1)	513 (38.3)	689 (52.2)	892 (64.1)
Validation cohort (n = 2,695)	IMRT alone	270 (39.1)	51 (7.5)	36 (5.8)	46 (6.5)
	CCRT	305 (44.2)	351 (51.3)	240 (38.8)	194 (27.6)
	IC+CCRT	115 (16.7)	282 (41.2)	342 (55.3)	463 (65.9)

= induction chemotherapy.

© 2020 Zhang LL et al. *JAMA Network Open.*

eFigure 1. Forest Plot Showing Univariate Cox Proportional Hazard Regression Analysis of Potential Risk Factors for Overall Survival in Nonmetastatic Nasopharyngeal Carcinoma

	HR (95%CI)	*P* value
**Age (years)**		
Training cohort		< 0.001
Validation cohort		< 0.001
**< 18 vs. ≥60**		
Training cohort	0.584 (0.258,1.321)	0.196
Validation cohort	0.323 (0.079,1.313)	0.114
**18–29 vs. ≥60**		
Training cohort	0.348 (0.243,0.497)	< 0.001
Validation cohort	0.260 (0.154,0.440)	< 0.001
**30–39 vs. ≥60**		
Training cohort	0.359 (0.282,0.456)	< 0.001
Validation cohort	0.342 (0.247,0.474)	< 0.001
**40–49 vs. ≥60**		
Training cohort	0.411 (0.333,0.509)	< 0.001
Validation cohort	0.376 (0.280,0.505)	< 0.001
**50–59 vs. ≥60**		
Training cohort	0.549 (0.440,0.686)	< 0.001
Validation cohort	0.396 (0.287,0.546)	< 0.001
**Gender (Female vs. Male)**		
Training cohort	0.730 (0.608,0.876)	0.001
Validation cohort	0.704 (0.540,0.918)	0.010
**WHO histologic type (Type III vs. Type I-II)**		
Training cohort	0.647 (0.446,0.939)	0.022
Validation cohort	0.593 (0.353,0.996)	0.048
**T stage**		
Training cohort		< 0.001
Validation cohort		< 0.001
**(T2 vs. T1)**		
Training cohort	1.742 (1.243,2.441)	0.001
Validation cohort	2.675 (1.616,4.428)	< 0.001
**(T3 vs. T1)**		
Training cohort	2.256 (1.698,2.996)	< 0.001
Validation cohort	2.980 (1.178,4.705)	< 0.001
**(T4 vs. T1)**		
Training cohort	4.137 (3.087,5.544)	< 0.001
Validation cohort	4.782 (1.387,7.618)	< 0.001
**N stage**		
Training cohort		< 0.001
Validation cohort		< 0.001
**(N1 vs. N0)**		
Training cohort	2.381 (1.718,3.300)	< 0.001
Validation cohort	1.729 (1.161,2.573)	0.007
**(N2 vs. N0)**		
Training cohort	4.118 (2.940,5.767)	< 0.001
Validation cohort	2.703 (1.778,4.108)	< 0.001
**(N3 vs. N0)**		
Training cohort	6.048 (4.281,8.543)	< 0.001
Validation cohort	3.980 (2.583,6.132)	< 0.001
**Cigarette consumption (Yes vs. No)**		
Training cohort	1.413 (1.215,1.644)	< 0.001
Internal validation cohort	1.407 (1.135,1.745)	0.002
**Alcohol consumption (Yes vs. No)**		
Training cohort	1.166 (0.915,1.428)	0.139
Internal validation cohort	1.256 (0.935,1.688)	0.130
**Family of cancer history (Yes vs. No)**		
Training cohort	0.968 (0.817,1.147)	0.705
Internal validation cohort	0.743 (0.575,0.962)	0.024
**EBV DNA (copy/mL) (≥ 2000 vs. < 2000)**		
Training cohort	2.645 (2.242,3.121)	< 0.001
Internal validation cohort	2.809 (2.220,3.556)	< 0.001
**HGB (g/L) (≥120 vs. <120)**		
Training cohort	0.803 (0.607,1.063)	0.125
Internal validation cohort	0.673 (0.470,0.964)	0.031
**LDH (U/L) (≥245 vs. <245)**		
Training cohort	2.046 (1.646,2.544)	< 0.001
Internal validation cohort	2.322 (1.696,3.178)	< 0.001
**ALB (g/L) (≥40 vs. <40)**		
Training cohort	0.556 (0.446,0.693)	< 0.001
Internal validation cohort	0.527 (0.389,0.714)	< 0.001
**CRP (mg/L)**		
Training cohort		< 0.001
Validation cohort		0.002
**(1.0-3.0 vs <1.0)**		
Training cohort	1.209 (0.997,1.466)	0.054
Internal validation cohort	1.272 (0.964,1.678)	0.089
**(≥3.0 vs <1.0)**		
Training cohort	1.718 (1.422,2.076)	< 0.001
Internal validation cohort	1.627 (1.237,2.140)	0.001

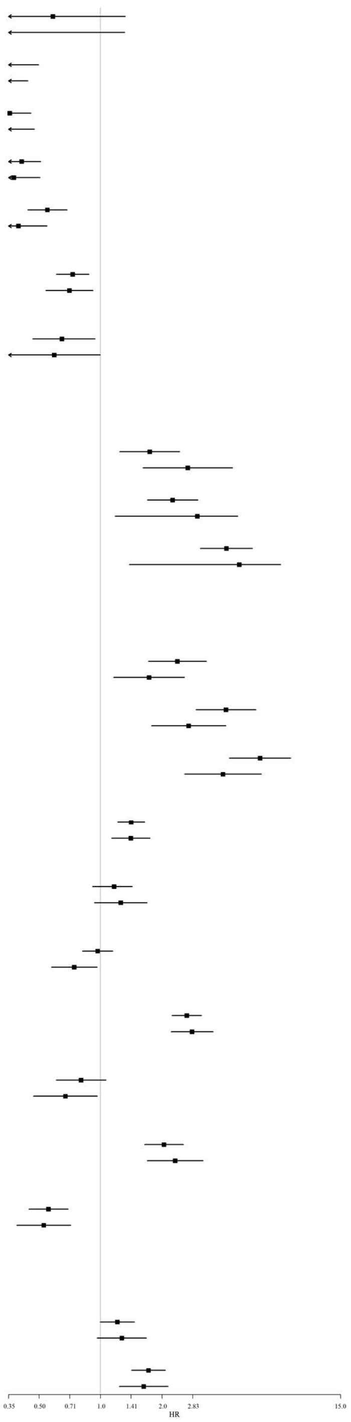

Abbreviations: WHO = World Health Organization; EBV DNA = Epstein-Barr virus; deoxyribonucleic acid; HGB = hemoglobin; LDH = lactate dehydrogenase; ALB = albumin; CRP = C-reactive protein.

© 2020 Zhang LL et al. *JAMA Network Open.*

eFigure 2. Receiver Operating Characteristic (ROC) Curves

Receiver operating characteristic (ROC) curves were developed to assess the performance of the proposed nomogram, TNM stage, and six single variables estimating overall survival for non-metastatic nasopharyngeal carcinoma in the training cohort (**A**, n = 5,398), and then validated in the validation cohort (**B**, n = 2,695). **Abbreviations:** AUC, area under the receiver operator characteristic curve.

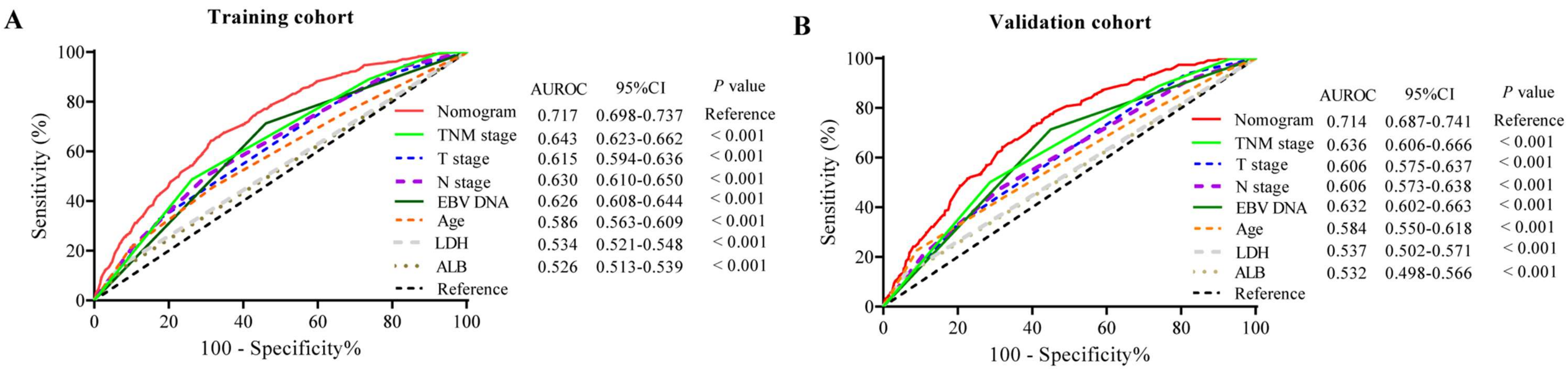

© 2020 Zhang LL et al. *JAMA Network Open.*
